# Coordination and Collective Performance: Cooperative Goals Boost Interpersonal Synchrony and Task Outcomes

**DOI:** 10.3389/fpsyg.2016.01462

**Published:** 2016-09-27

**Authors:** Jamie S. Allsop, Tomas Vaitkus, Dannette Marie, Lynden K. Miles

**Affiliations:** ^1^School of Psychology, University of AberdeenAberdeen, UK

**Keywords:** interpersonal synchrony, cooperation, competition, productivity, teamwork, coordination, groups

## Abstract

Whether it be a rugby team or a rescue crew, ensuring peak group performance is a primary goal during collective activities. In reality, however, groups often suffer from productivity losses that can lead to less than optimal outputs. Where researchers have focused on this problem, inefficiencies in the way team members coordinate their efforts has been identified as one potent source of productivity decrements. Here, we set out to explore whether performance on a simple object movement task is shaped by the spontaneous emergence of interpersonally coordinated behavior. Forty-six pairs of participants were instructed to either compete or cooperate in order to empty a container of approximately 100 small plastic balls as quickly and accurately as possible. Each trial was recorded to video and a frame-differencing approach was employed to estimate between-person coordination. The results revealed that cooperative pairs coordinated to a greater extent than their competitive counterparts. Furthermore, coordination, as well as movement regularity were positively related to accuracy, an effect that was most prominent when the task was structured such that opportunities to coordinate were restricted. These findings are discussed with regard to contemporary theories of coordination and collective performance.

## Introduction

Many of life’s most valued outcomes are only attainable by combining efforts with others. No amount of exertion, or expertise, will ever allow the lone rugby player to defeat an opposition team of 15. Similarly, achievements in a modern operating theater, flight deck, boardroom, or restaurant kitchen are enabled to the extent that individual agents act in concert with colleagues. Teamwork, however, is not all moonlight and roses. Not only can group performance exceed the capacity of individual members, but teams can also underperform by failing to optimally realize their collective potential. While researchers have identified several phenomena that characterize specific aspects of group productivity (e.g., social loafing, social facilitation, Köhler effect), the issue, in essence, is one of coordination. Combining efforts leads to the emergence of dependencies (i.e., links) between team members. The efficiency of these links, that is, the extent to which each member’s actions are functionally coordinated, in large part determines the effectiveness of the group.

Grounded in an extensive literature concerning collective performance (see Kozlowski and Ilgen, 2006 for an overview), contemporary theorists have argued that teamwork can be conceptualized as a complex dynamical system (e.g., McGrath et al., 1999; Marks et al., 2001; Gorman et al., 2010; Waller et al., 2016). Specifically, rather than characterize group productivity as the simple aggregate of each member’s individual level attributes (e.g., *a priori* skill, motivation, capacity), the dynamical stance proposes that collective performance is an emergent property, arising from the interaction of the system’s components over time (Kelso, 1995; Schmidt and Richardson, 2008). Viewed in this way, patterns of productivity are not determined by top-down linear cause-and-effect relationships, but instead emerge via the intermittent and non-linear interactions between individual team members. The effectiveness of, for instance, a team consisting of a rally driver and navigator is not a linear combination of their respective skill levels — excellent navigation combined with poor driving is unlikely to yield performance equivalent to similarly excellent driving paired with poor navigation. In other words, team performance can be considered to emerge from the quality of the functionally specific interactions between team members, that is, the degree to which task-relevant dependencies are coordinated.

What then, does it mean to be coordinated in this sense? Conceptually speaking the teamwork literature considers coordination to encapsulate the range of activities (e.g., goal sharing, task assignment, resource allocation) required to effectively manage the timing and execution of interdependent efforts within a group (Steiner, 1972; Marks et al., 2001; Kozlowski and Bell, 2003; Espinosa et al., 2004). Although broad and clearly context-specific, two key commonalities have been identified that constitute coordinated efforts (Kozlowski and Ilgen, 2006). Coordination involves: (i) the integration of distinct actions; (ii) in a manner that is temporally aligned with other contributions. Typically, coordination in applied team settings is thought to come about via learning, experience, and expertise, and is managed via both explicit (e.g., instruction) and implicit (e.g., tacit understanding) mechanisms (Espinosa et al., 2004). However, core aspects of this approach are grounded in social-cognitive models, demanding voluminous information processing and top-down control (Araújo and Bourbousson, 2016). Construed in this way, coordination-driven teamwork then becomes an arguably impossible (Turvey, 1990, 2007; Turvey and Fonseca, 2009) achievement of individual minds, rather than an emergent property of the interactions between team members. In contrast, the science of coordination dynamics (e.g., Kelso, 1995) posits that coordination is self-organizing, emerging spontaneously precisely because of the interactions between individual components of a system (e.g., team members). Adopting this approach may therefore provide a more theoretically tractable framework for understanding how coordination impacts collective productivity.

Inspired by centuries-old observations of spontaneous alignment in mechanical devices (e.g., pendulum clocks; Huygens, 1673/1986), the lawful principles of coordination dynamics indicate that components of systems which are both coupled (i.e., linked) and share specific qualities (e.g., movement frequency), will tend to spontaneously synchronize (i.e., coordinate in time^1^) toward one of two attractor states (i.e., in-phase or anti-phase; Kelso, 1995). Indeed, these specific patterns have been documented in many biological systems, ranging from fields of fireflies (e.g., Buck and Buck, 1976) to people in social contexts (e.g., Schmidt and O’Brien, 1997; Richardson et al., 2007b). Importantly, interpersonal coordination brings with it a host of socially relevant outcomes that function to establish a common ground and enhance entitativity (Semin, 2007; Schmidt and Richardson, 2008; Marsh, 2013). For instance, even short periods of synchronous action promote affiliation (Hove and Risen, 2009) and cooperation (Wiltermuth and Heath, 2009) between interaction partners, while negative social contexts have been shown to thwart the emergence of synchrony (Miles et al., 2010; Paxton and Dale, 2013a).

Demonstrations of spontaneous interpersonal coordination are plentiful (see Marsh, 2013 for an overview). Not only do people unintentionally align their gross motor behavior (e.g., footsteps; Zivotofsky and Hausdorff, 2007) but also their gaze (Richardson and Dale, 2005), speech patterns (Fusaroli et al., 2012), postural movements (Shockley et al., 2003), and heart rate (Mitkidis et al., 2015), to name but a few examples. Acknowledging the enormous computational burden demanded by representational explanations of joint action,^2^ researchers have recently highlighted how insight into the dynamics of interpersonal activity may provide more parsimonious accounts of collective behavior (Schmidt and Richardson, 2008; Coey et al., 2012; Dale et al., 2013). To illustrate, Richardson et al. (2015) investigated the behavioral dynamics of a goal-directed joint targeting task. Pairs of participants repetitively moved virtual objects to target locations with the instruction to avoid collisions. Importantly, the set-up was such that if both participants followed the optimal movement trajectory (i.e., a straight line) they would collide and fail to complete the task. The data revealed that, without communication, participants rapidly and spontaneously adopted an asymmetric pattern of movement with one maintaining the direct trajectory, while the movements of the other showed a more elliptical shape. Dynamical modeling supported this observation whereby a between-participant asymmetry in repeller (i.e., collision avoidance) strength reflected the behavioral data. Here then, participants were seen to spontaneously adapt their movements relative to one another in a manner functionally consistent with task-relevant dependencies (i.e., move objects and avoid colliding). Crucially, in line with a dynamical systems approach, the adoption of asymmetrical but complementary roles (i.e., one straight and one elliptical trajectory) emerged naturally from the interactions between participants and task constraints, rather than from any top-down, *a priori* plan or set of instructions.

A rapidly growing body of work attests to the notion that patterns of movement that can characterize self-organized interpersonal coordination are also implicated in effective joint performance. For instance, Abney et al. (2015) reported that performance on a joint tower-building task was improved when partners’ body movements were loosely coupled. Although assigned to distinct task-specific roles and being freely available to communicate, pairs who displayed moderate levels of motor coordination also constructed better towers. Similarly, Fusaroli et al. (2016) showed that uninstructed behavioral coordination positively predicted competence in a group LEGO^®^ building task, while Won et al. (2014) reported that dyads tasked with idea generation were more creative to the extent that they synchronized their movement. More concrete joint action tasks also reveal a functional role for spontaneous motor coordination. People readily make very fine-grained adjustments to their behavior and spontaneously take on distinct task-relevant roles in order to achieve coordination goals (e.g., coordinating landing times when jumping; Vesper et al., 2013). In seminal demonstrations, when given the exercise of moving planks of differing lengths without verbal communication, pairs of participants adopt different behavioral modes (i.e., one-handed, two-handed, or two-person lifting) depending on both plank length and partner ability (Richardson et al., 2007a; Isenhower et al., 2010). Together, what this work indicates is that beyond the notion that people can (and do) coordinate their actions with others, functional task-specific patterns of coordination emerge from goal-oriented interactions — a key characteristic of a self-organizing social system.

The current research sought to further explore the notion that collective performance can be understood in the terms of a self-organized dynamical system. By focusing on an ecologically-relevant outcome of group work – productivity – we aimed to identify whether performance in this sense is influenced by the spontaneous emergence of interpersonally coordinated behavior. Participants, both individually and as a pair, were asked to move small plastic balls from one location to another as quickly and accurately as possible. We manipulated two factors intended to shape the nature of the task-relevant dependencies (i.e., links) between individuals. First, as a within-participants factor, we adjusted the aperture of the target location (i.e., where the balls were deposited) so that either only one ball (i.e., small aperture condition) or two balls (i.e., large aperture condition) could be deposited at a time. In effect, this varied the affordances (i.e., opportunities for action; Gibson, 1979) available to participants and, in turn, the possibilities for coordination. Specifically, the potential for in-phase coordination (i.e., 0° relative phase, both participants pick-up and deposit balls simultaneously) was eliminated in the small aperture condition. Second, we varied the social context in a between-participants manner by manipulating the instructional set – either to compete or cooperate – given to each pair. This factor was intended to influence performance-related dependencies between participants to the extent that cooperative goals promote interdependent modes of action, while competitive goals lead to more independent behavior (Deutsch, 1949; Beersma et al., 2003).

By manipulating task-relevant dependencies, we created a context in which both productivity and coordination were expected to vary in systematic ways. For each trial we quantified productivity in terms of both the number of balls successfully transferred (i.e., hits) and the number dropped (i.e., misses). We expected the small (cf. large) aperture condition to limit productivity, resulting in fewer hits and more misses. Similarly, consistent with Beersma et al. (2003), we expected the cooperation/competition instructions to result in a form of a speed-accuracy trade-off, leading to ‘co-operators’ being more accurate (i.e., fewer misses) and ‘competitors’ more productive (i.e., more hits). We also tracked each participant’s actions using a video-based frame-differencing approach (Schmidt et al., 2012; Paxton and Dale, 2013b; Romero et al., 2016) and used the resulting time-series to estimate movement variability and between-participant coordination. Here we expected to see evidence of interpersonal coordination and an accompanying reduction in movement variability (i.e., increased stability), but this to be tempered by aperture size (i.e., small aperture to reduce levels of coordination as the in-phase mode is not possible) and instructions to compete (i.e., resulting from the reduction in interdependency). With these predictions in mind, we also set out to begin to address a more overarching question — what is the relationship between movement coordination and group productivity?

## Materials and Methods

### Participants and Design

In total, 102 undergraduate participants took part in pairs in return for course credit. However, prior to analysis, five pairs were removed from the dataset on the basis that participants reported knowing each other.^3^ The final sample consisted of 92 participants (72 female, age range 17–35 years, mean age = 20.7 years). The study had a three-factor mixed model design whereby task context (solo vs. group) and aperture size (small vs. large) were manipulated within participants, while instruction set (cooperation vs. competition) was manipulated between participants (i.e., 23 pairs per condition). The study was reviewed and approved by the School of Psychology, University of Aberdeen ethics committee.

### Materials and Procedure

**FIGURE 1:**
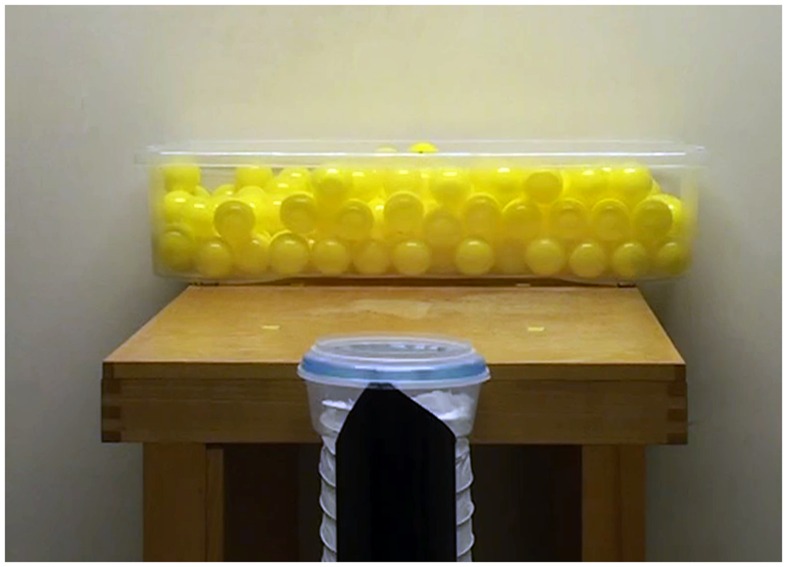
**The object movement task set-up (large aperture condition)**.

Pairs of participants arrived at the laboratory individually and were briefly introduced to each other before being separated into adjacent rooms. At this point, one participant completed questionnaires to provide basic demographic information (see Supplemental Materials) while the other was introduced to the object movement task. The task (see **Figure 1**) required participants to move small plastic balls (6 cm diameter), one at a time, from a large container (75 cm x 35 cm), fixed to the top of a table, to a tube located approximately 110 cm away. The receptacle tube was fitted with a lid with an aperture of either 7.5 cm (small aperture condition) or 15.5 cm (large aperture condition). The order of tube size was counterbalanced across pairs. Participants were required to use their dominant hand only while keeping their other hand behind their back, and to move each ball using a single arm movement without throwing them (i.e., to drop or place them into the tube). Importantly, participants were instructed to move the balls as quickly and accurately as possible.

Initially, participants completed 4 trials individually. Two trials were completed for each aperture size, one from each side of the table, and data were averaged across these trials. Once the first participant had completed this stage, they swapped rooms and filled out the demographic items while the other participant performed the object movement task. Each trial lasted for 65 s and was preceded by a 3 s countdown. Participants were given the option of a short break at the end of each trial if they were fatigued in any way. Immediately after both participants had completed the individual trials, they were invited back to the main laboratory to perform the task again, but this time together as a dyad. Participants were randomly assigned to either the cooperative or competitive conditions and at this point were given their instructions. Specifically, those in the cooperative condition were told to: “move the balls as quickly and accurately as possible, as a pair. That is, you need to cooperate with each another in order to achieve the goal.” In contrast, those in the competitive condition were instructed to: “move the balls as quickly and accurately as possible, as an individual. That is, you need to compete against each other in order to achieve the goal.” All participants were also instructed to not verbally communicate with each other. Again, each pair completed 4 trials, two for each aperture size, one from each side of the table. All trials were recorded to video (1920 px × 1080 px, 25 fps) using a digital video camera (Sony HD-SR12). Care was taken to ensure the camera was aligned with the center of the table/receptacle tube in order to be able to isolate each participant’s movements (see Romero et al., 2016). After completing all trials participants were thanked for their time, debriefed, and dismissed.

### Data Reduction and Analysis

**FIGURE 2:**
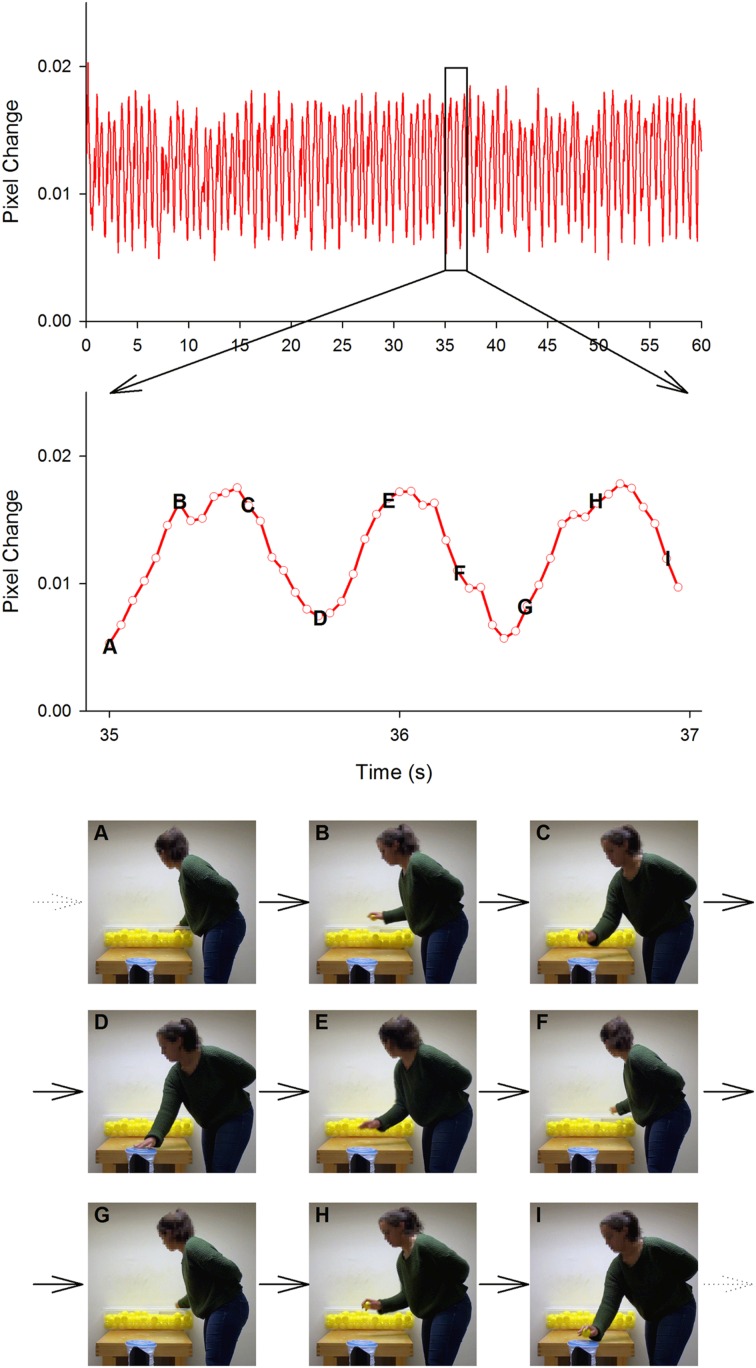
**Illustration of the frame-differencing technique used to quantify movement**. A full 60 s time-series of movement (i.e., pixel change) from a solo trial is shown in the top panel and a ‘zoomed’ 2 s (35 s – 37 s) period in the middle panel. The lower panels depict every 6th frame (≈ ¼ s) from this 2 s period. The letter on each frame denotes the corresponding data point on the ‘zoomed’ time-series. As can be seen, the oscillatory pattern of the time-series data corresponds to the participant’s actions. ‘Valleys’ (i.e., low amount of movement/pixel change) match either picking up a ball from the container (e.g., frame A) or depositing it in the tube (e.g., frame D), while ‘peaks’ (i.e., high amount of movement/pixel change) match periods of movement between container and tube (e.g., frame B).

Prior to analysis, the first 5 s of each trial was truncated in order to remove the countdown period and to eliminate any initial transient movements. A frame-differencing approach was then employed using a custom-written MATLAB script to convert the remaining 60 s of each trial into movement time-series. Specifically, each frame was halved vertically (in order to separate each participant’s movements) and compared to the corresponding half of the previous frame in terms of pixel change (see **Figure 2**). This provided two time-series (one per participant) of movement data for each trial (one time-series for individual trials).

Global movement coordination was quantified using cross-spectral coherence (Porges et al., 1980; Gottman, 1981; Warner, 1988). Each time-series was submitted to a cross-spectral analysis and expressed as component frequencies before the correlation between the two time-series (in the frequency domain) was calculated as a weighted average across the component frequency range. This measure provided an estimate of the extent to which participants’ actions were temporally aligned (with 0 representing no movement coordination and 1 representing complete movement coordination) and has been commonly employed as an index of interpersonal coordination (e.g., Sadler et al., 2009; Lumsden et al., 2012; Schmidt et al., 2014). For each time-series we also calculated the coefficient of variation (CV) as an index of movement variability. For this measure we initially calculated the mean and standard deviation of the period (i.e., distance between ‘peaks’ on each time-series) for each participant on each trial individually. Analysis revealed that the mean period differed as a function of aperture size and task context, hence rather than raw standard deviation we used the coefficient of variation (CV = σ/μ) as a standardized index of the temporal regularity of participant movements (i.e., higher CV values = less regular movements). Finally, we also recorded the number of hits and misses per participant per trial for the same specific 60 s period from which the movement time-series were constructed.

**FIGURE 3:**
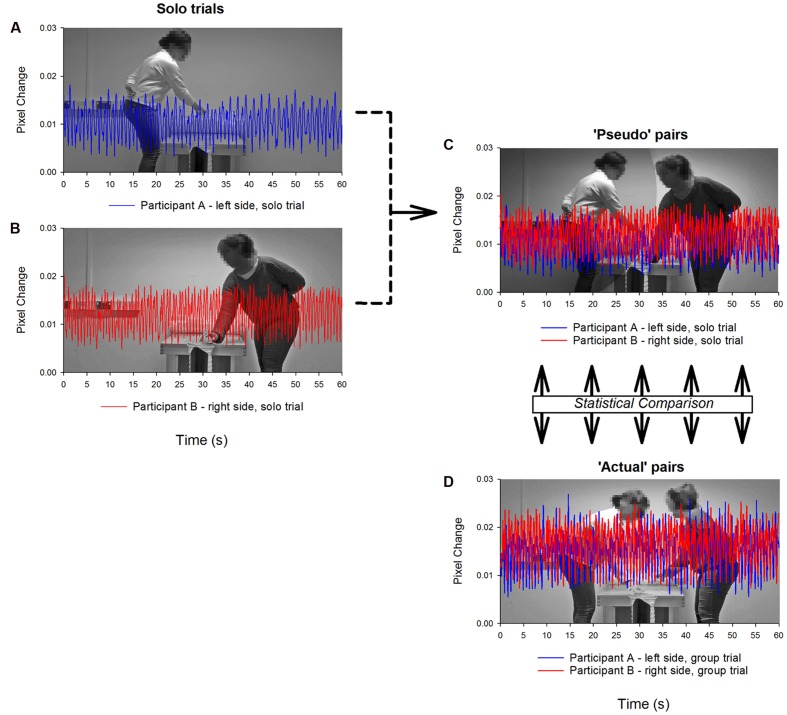
**Illustration of the procedure for constructing pseudo-pairs**. Data (i.e., movement time-series, task performance) from each participant’s solo trials **(A,B)** is combined **(C)** and compared to the equivalent joint trial **(D)**.

To provide an estimate of baseline performance we constructed pseudo-pairs by combining data from relevant individual trials (see **Figure 3**). For example, for a given trial (e.g., small aperture), data from the first participant’s individual trial from the left side of the table were combined with that from the second participant’s individual trial from the right side of the table. This provided baseline data specific to each pair in terms of expected performance (i.e., should their group-level productivity be a simple linear combination of their individual efforts), as well as an estimate of incidental (i.e., chance) levels of coordination. Therefore, across all measures the unit of analysis was at the level of the dyad.

## Results

Initially, the primary dependent variables were analyzed separately using 2 (pair type: pseudo vs. actual) × 2 (aperture size: small vs. large) × 2 (instructions: cooperation vs. competition) mixed model analysis of variance (ANOVA) with repeated measures on the first two factors. Significant effects are reported below.

### Productivity: Hits

**FIGURE 4:**
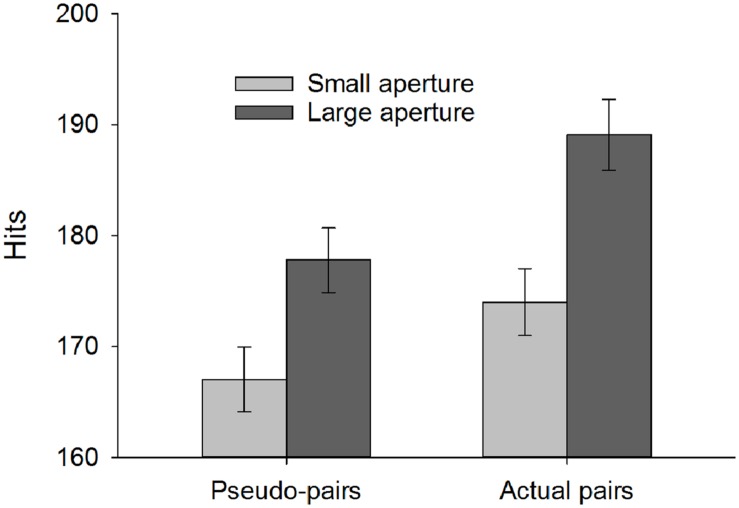
**Hits (i.e., balls successfully deposited) as a function of pair type and aperture size**. Error bars represent ±1 SEM.

With respect to the number of balls successfully deposited, the analysis revealed main effects of both pair type, *F*(1,44) = 34.03, *p* < 0.001, $\eta_{p}^{2}$ = 0.44 (i.e., pseudo < actual), and aperture size, *F*(1,44) = 178.12, *p* < 0.001, $\eta_{p}^{2}$ = 0.80 (i.e., small < large), which were qualified by an interaction between these factors, *F*(1,44) = 6.87, *p* = 0.012, $\eta_{p}^{2}$ = 0.14, as shown in **Figure 4**. *Post hoc* pairwise comparisons (Bonferroni corrected) confirmed that actual pairs were more productive than would be expected by combining their solo efforts (i.e., pseudo-pairs) for both the small (*p* < 0.001) and large (*p* < 0.001) apertures.

### Productivity: Misses

**FIGURE 5:**
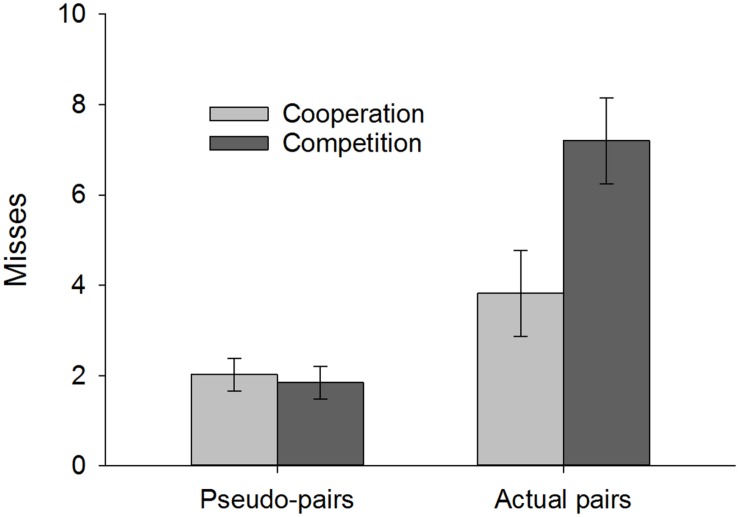
**Misses (i.e., balls dropped) as a function of pair type and instruction condition**. Error bars represent ±1 SEM.

When considering the number of balls dropped or missed, the analysis revealed a main effect of pair type, *F*(1,44) = 34.57, *p* < 0.001, $\eta_{p}^{2}$ = 0.44 (i.e., pseudo < actual), and a marginally significant effect of condition, *F*(1,44) = 3.82, *p* = 0.057, $\eta_{p}^{2}$ = 0.08 (i.e., cooperation < competition), which were qualified by an interaction between these factors, *F*(1,44) = 8.49, *p* = 0.006, $\eta_{p}^{2}$ = 0.16, as shown in **Figure 5**. *Post hoc* pairwise comparisons (Bonferroni corrected) indicated that while there was no difference as a function of condition when solo efforts (i.e., pseudo-pairs) were combined (*p* = 0.74), actual pairs in the competitive condition made significantly more errors (i.e., more misses) than those in the cooperative condition (*p* = 0.03).

### Movement Coordination

**FIGURE 6:**
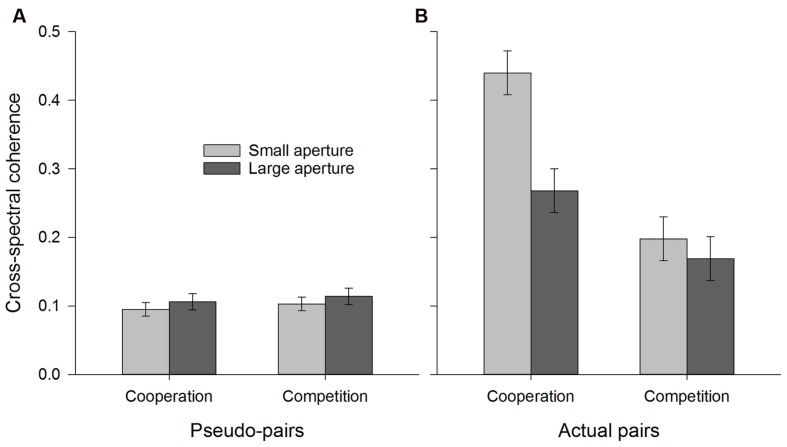
**Coordination (i.e., cross-spectral coherence) as a function of instruction condition and aperture size**. **(A)** represents pseudo-pairs and **(B)** represents actual pairs. Error bars represent ±1 SEM.

Analysis of coordination (i.e., cross-spectral coherence) revealed that all main effects and 2-way interactions reached significance (all *F*s>5.8) and were ultimately qualified by a 3-way interaction between pair type, aperture size, and condition, *F*(1,44) = 5.80, *p* = 0.02, $\eta_{p}^{2}$ = 0.12, as shown in **Figure 6**. To simplify interpretation we then conducted separate 2 (aperture size: small vs. large) × 2 (instructions: cooperation vs. competition) mixed model ANOVAs for the pseudo-pairs (**Figure 6A**) and actual pairs (**Figure 6B**) separately. As expected, for the pseudo-pairs there were no significant effects (all *F*s < 1), indicating that incidental (i.e., chance) levels of coordination were equivalent across conditions and aperture size. In contrast, for actual pairs there were main effects of both aperture size, *F*(1,44) = 13.51, *p* = 0.001, $\eta_{p}^{2}$ = 0.24 (i.e., small > large), and instructions, *F*(1,44) = 22.38, *p* < 0.001, $\eta_{p}^{2}$ = 0.34 (i.e., cooperation>competition), which were qualified by an interaction between these factors, *F*(1,44) = 6.88, *p* = 0.012, $\eta_{p}^{2}$ = 0.14. *Post hoc* pairwise comparisons (Bonferroni corrected) indicated that for participants who had been instructed to cooperate, levels of coordination were higher when depositing balls into the small aperture tube compared to the large one (*p* = 0.006), while there was no such difference for those in the competitive condition (*p* = 0.34).

### Movement Variability

Comparison of the CV indicated main effects of aperture size, *F*(1,44) = 54.73, *p* < 0.001, $\eta_{p}^{2}$ = 0.55 (i.e., small < large), and pair type, *F*(1,44) = 81.41, *p* < 0.001, $\eta_{p}^{2}$ = 0.65 (i.e., pseudo < actual). Movements were more regular when depositing into the small tube, and when performing the task alone.

### Coordination, Movement Variability, and Task Performance

Finally, we examined the simple linear relationship between the level of coordination that emerged between each pair, movement variability, and productivity levels (i.e., hits and misses separately) for each aperture size. As displayed in **Table 1**, when considering the small aperture there was a clear negative relationship between coordination and accuracy (i.e., misses), *r*(46) = -0.41, *p* = 0.004, whereby pairs whose actions were more coordinated were also more accurate (i.e., fewer misses). Similarly, pairs who showed less variability in their movements also made fewer errors when depositing into the small aperture, *r*(46) = 0.42, *p* = 0.004. We then entered both the coordination and variability measures (from the small aperture condition) as predictors into a multiple regression analysis with accuracy (i.e., misses) as the outcome variable of interest. The overall model was significant, *F*(2,45) = 10.13, *p* < 0.001, and accounted for approximately 30% of the variance (adjusted *R*^2^ = 0.289). Importantly, both variables were seen to be independent significant predictors of accuracy: coordination, *β* = -0.38, *t*(43) = -3.03, *p* = 0.004; variability, *β* = 0.39, *t*(43) = 3.07, *p* = 0.004.

**Table 1:** Correlations between coordination (i.e., cross-spectral coherence), movement variability (i.e., coefficient of variation [CV]), hits, and misses for the small and large apertures separately.

l	Coordination	Variability	Hits	Misses
**Small aperture (*n* = 46)**				
lCoordination	—	-0.08	-0.10	-0.41^a^
lVariability		—	0.17	0.42^a^
lHits			—	-0.19
lMisses				—
**Large aperture (*n* = 46)**				
lCoordination	—	-0.03	-0.14	-0.22
lVariability		—	0.12	0.22
lHits			—	-0.08
lMisses				—

*A matrix of scatterplots depicting these relationships is provided in the Supplemental Materials.*^a^*p = 0.004*

On the other hand, when depositing the balls into the large aperture there were no significant relationships between any of the factors. However, inspection of **Table 1** suggests these effects were consistent in terms of direction but reduced in magnitude compared with those found for the small aperture.

## Discussion

The present results provided support for the predicted effects, but also revealed some unanticipated outcomes. Importantly, here we demonstrated that in the context of a simple object movement task, the presence of a co-actor led to facilitated productivity (i.e., more hits) and a decrease in accuracy (i.e., more misses) beyond the extent that would be expected by simply combining solo efforts. Characteristic of classic ‘social facilitation’ effects (e.g., Triplett, 1898; Zajonc, 1965), the product of working collectively exceeded the sum of the individual inputs. Similarly, across all conditions, coordination was greater than would be expected had each individual not been impacted by the presence of the other (i.e., chance). Together these findings point to the notion that performance at the dyadic level emerged from the real-time interactions between the participants and the environment, rather than simply being the linear product of each individual’s *a priori* attributes and static task constraints. This view lends further support to the notion that group productivity can be conceptualized as an emergent phenomenon (McGrath et al., 1999; Marks et al., 2001; Gorman et al., 2010; Waller et al., 2016).

When it came to the relationship between task performance and movement the results provide further insight into the functional aspect of this connection. Here, it could be expected that more is simply better — that stable coordinative states best realize between-participant dependencies and in turn facilitate greater productivity. The data, however, suggest a different, potentially more nuanced situation (cf. Abney et al., 2015). Both of the movement-relevant measures we considered (i.e., variability and coordination) were seen to exert influence on task performance, but primarily in terms of shaping accuracy rather than gross productivity. Pairs that showed higher levels of coordination *or* more regular movements also tended to be more accurate (i.e., fewer misses). The effects on hits were similar in directional terms but did not reach significance. Of note, there was no relationship between the measures of coordination and movement variability, suggestive of these factors having distinct roles in shaping task performance. Thus, it appears that in the context of the current task, the emergence of interpersonal coordination, along with regular movement patterns, were associated with more accurate performance.

Where these effects were most robust, both coordination levels and movement regularity independently predicted task accuracy when participants were depositing balls into the small aperture. Moreover, this condition was seen to result in the lowest level of productivity but the highest level of coordination. Although speculative, we suggest that the restriction of the small aperture led participants to fall into an anti-phase mode of coordination (i.e., when one participant is picking up a ball the other is depositing).^4^ Acknowledging that this mode of coordination is stable at relatively lower movement frequencies (Haken et al., 1985; Kelso, 1995; Schmidt and Richardson, 2008), it follows that this slowing, in combination with the physical spacing of participants’ actions (i.e., in an anti-phase mode, collisions at the pick-up and depositing regions are effectively eliminated) could result in the heightened accuracy observed. Relatedly, if there were fewer collisions between participants, this may also explain the effects of movement variability in that these instances will necessarily perturb regular rhythmic movements as participants recover and adjust their actions accordingly. Quite why participants appeared to avoid the globally stable in-phase mode of coordination when available (i.e., large aperture) is, however, unclear.^5^ Imperative, therefore, is for future work to seek to employ more precise methods (e.g., high fidelity motion-tracking) to better capture the dynamical characteristics of instances of coordination as reported here.

Two additional findings merit consideration. First, while there is a solid evidential basis to suggest that engaging in synchronous acts can promote subsequent cooperative behavior (e.g., Wiltermuth and Heath, 2009; Valdesolo et al., 2010; Kokal et al., 2011; Launay et al., 2013; Reddish et al., 2013; Cirelli et al., 2016), work addressing the converse relationship – cooperation engendered synchrony – is scarce. Although it has previously been established that individuals with pro-social motives show higher levels of spontaneous interpersonal synchrony (Lumsden et al., 2012), to our knowledge the current study provides the first empirical demonstration that an explicit instruction to cooperate (cf. compete) also leads to a greater tendency to coordinate behavior. Our findings point toward a bidirectional relationship between coordination and cooperation. We believe this adds weight to the claim that this association may operate as a feedback loop — establishment of coordination has been argued to provide immediate real-time reinforcement for cooperative intentions, which in turn support further coordination (Reddish et al., 2013). In a related sense, those instructed to compete in the current study not only showed reduced levels of coordination, they also made more errors. As well as contributing support for the speed-accuracy trade-off documented by Beersma et al. (2003), this effect may again reflect a reinforcement of behavior over time. If competitive motives initially thwart the emergence of coordination, this may function to simply maintain the state of affairs which, in the context of the current task, was seen to result in decreased accuracy. Future work focused on developing a more fine-grained understanding of the real-time evolution of the relationship between coordination and productivity will help further evaluate this proposal.

Consideration of limitations of the current study also warrants some attention. First, we acknowledge that by always testing the solo performance condition first, we are unable to eliminate the potential influence of practice or carry-over effects. However, in the present task it was important to initially establish a baseline individual performance level free of any ‘social contamination’ (e.g., from observing a partner’s performance), an approach that is also employed in related literature (e.g., Vesper et al., 2013). Moreover, given all participants performed their solo trials under identical conditions (i.e., prior to the cooperation-competition instruction set) it would be reasonable to expect any practice effects to be consistent across conditions. The motor task itself is also very straightforward, suggesting practice might be of limited benefit. Nonetheless, it is of course important for future work to empirically investigate this matter by counterbalancing the order of individual and group trials. Second, although the present pattern of results is consistent with a self-organized dynamical account of interpersonal coordination, we cannot effectively rule out more strategic socially-relevant behavior. For instance, participants may take more care when in the cooperative (cf. competitive) condition so as to limit the impact of their errors on their partner, intentionally take on a complementary role to their partner (cf. Vesper et al., 2013; Richardson et al., 2015), or even simply show ‘good manners’ by, for instance, pausing to allow their partner to proceed. Examining participant strategies, by conducting qualitative interviews post performance, for example, may help provide additional insight here and improve the generality of the current findings.

Along with furthering the empirical understanding of the functional relationships between coordination and collective activity, here we also outlined a novel object-movement task that we feel is well-suited for the experimental investigation of group dynamics. The current task is simple and inexpensive to run, allows for both laboratory and field settings, and has ample scope for ‘scaling-up’ to multi-agent activities. The task provides a procedure to establish meaningful baseline estimates of group behavior (i.e., pseudo-groups) and enables precise quantification of such behavior, while allowing participants to behave in a relatively naturalistic fashion. To this end, the present results provide some proof-of-concept that the task is a suitable vehicle for studying the effects of both social and physical parameters. Further validation of the task in combination with the introduction of more detailed behavioral recording (i.e., high fidelity motion tracking) are, therefore, important next steps.

More broadly, the current study also contributes to an increasingly complex picture regarding the general relationship between interpersonal synchrony and collaborative activity. Although consistent with prominent claims that synchrony is a pervasive feature of social life (Schmidt and Richardson, 2008; Marsh, 2013), more detailed functional arguments are likely to demand greater context-specificity. That is, understanding precisely how and when interpersonal coordination functions to enhance goal-directed joint activity is likely to require a more systematic specification of how between-person task dependencies are best managed in order to optimize performance. Although clearly challenging, this approach may offer valuable insight into how we might structure group activity in order to best realize the potentials of teamwork.

## Author Contributions

All authors developed the study concept and design. JA and TV collected the data. JA, TV, and LM conducted the data analysis and interpretation of results. JA and LM drafted the manuscript and TV and DM provided critical review and revisions. All authors approved the final version of the manuscript for submission.

## Conflict of Interest Statement

The authors declare that the research was conducted in the absence of any commercial or financial relationships that could be construed as a potential conflict of interest.

## Footnotes

1 There is a variety of terminology used in the literature to describe interpersonal coordination (e.g., alignment, convergence, mirroring, mimicry, synchrony). Here, following Paxton and Dale (2013a; also see Lumsden et al., 2014), we equate ‘synchrony’ to behaviors that are matched in time and space (e.g., phase locked) and use, coordination, as a more general term to capture the range of non-spurious relationships between the behaviors of interacting individuals.

2 Critiques of information-processing and/or representational accounts typically focus on two general issues: (i) a gross excess of information to process/control (i.e., the *degrees-of-freedom* problem; Bernstein, 1967), and (ii) the absence of a plausible processor or executive controller (i.e., the *homunculus* problem; Ryle, 1949/2009). Although a detailed treatment of these arguments is beyond the scope of the present article, we point interested readers toward several excellent overviews (e.g., Reed, 1996; Richardson et al., 2008; Chemero, 2011).

3 Each participant indicated how well they knew the other by marking a vertical line on a 150 mm analog scale anchored by ‘Not at all’ and ‘Extremely well’. Pairs with an average familiarity rating >10 (i.e., 10 mm from ‘Not at all’) were excluded from the analysis.

4 Anecdotal observations recorded by the experimenters during the task are consistent with this conjecture. In addition, we conducted two follow-up procedures that also support this interpretation (see Supplemental Materials).

5 One speculative suggestion is that although physically sufficient, the size of the large aperture (i.e., ≈ 2.6 times the ball diameter) was still too restrictive to allow participants to comfortably deposit two balls simultaneously without touching hands, etc.
