# Supplementary material for: Coordination and Collective Performance: Cooperative Goals Boost Interpersonal Synchrony and Task Outcomes
Source: Front Psychol. 2016 Sep 27;7:1462. doi: 10.3389/fpsyg.2016.01462 (PMC5037181; doi:10.3389/fpsyg.2016.01462)
Supplement: Supplementary file 1 [file Presentation_1.PDF]

Coordination and collective performance:
Cooperative goals boost interpersonal synchrony and task outcomes.

Jamie S. Allsop, Tomas Vaitkus, Dannette Marie, & Lynden K. Miles
School of Psychology, University of Aberdeen, UK

Supplemental Materials

Participant Demographics

Prior to the group stage of the procedure we collected basic demographic information (i.e., participant age, sex, and handedness) along with assessments of select personality characteristics previously shown to relate to interpersonal synchrony (i.e., social value orientation, see Lumsden, Miles, Richardson, Smith, & Macrae, 2012; social anxiety, see Varlet et al., 2014; autism spectrum, see Fitzpatrick et al., 2016). As shown in Table S1 (categorical variables) and S2 (continuous variables) there were no systematic differences between conditions on any of the measures.

Table S1: Demographic and personality measures (categorical).

Variable	Condition				
	Cooperation	Competition			
	n	*n*	χ^2	df	*p*
Sex (individual)					
Female	35	37	0.25	1	.61
Male	11	9			
Sex (dyad)					
Same[a]	14	15	0.09	1	.76
Different	9	8			
Hand (individual)					
Right	40	37	0.72	1	.40
Left	6	9			
Hand (dyad)					
Same[b]	19	16	1.07	1	.30
Different	4	7			
SVO[c]					
Pro-social	23	20	0.72	2	.70
Pro-self	17	21			
No classification	6	5			

Notes: [a] There was one male-male pairing in the cooperative condition. Repeating the analysis without this pair yielded an identical result. [b] There were two pairs for which both participants were left-handed, one in each condition. Repeating the analysis without these pairs yielded an identical result. [c] Social Value Orientation (SVO) as measured by the 9-item triple dominance measure (see Van Lange, 1999). Here we combined the 'Individualist' and 'Competitive' orientations into a general 'Pro-self' category. 'No classification' refers to participants who failed to make at least 6/9 consistent (i.e., same orientation) choices. Repeating the analysis with the 'no classification' group excluded also yielded a non-significant result, $\chi^2(1, N = 81) = 0.62, p = .43$.

Table S2: Demographic and personality measures (continuous).

Variable	Condition				
	Cooperation	Competition			
	M (SD)	*M (SD)*	*t*	df	*p*
Age (years)	21.3 (4.1)	20.2 (2.3)	1.55	90	.13
LSAS[a]	49.6 (24.6)	47.0 (19.9)	0.57	90	.57
AQ[b]	18.2 (7.2)	16.5 (5.2)	1.31	90	.19

Notes: [a] Liebowitz Social Anxiety Scale (Liebowitz, 1987) total score (subscales, Cronbach's $\alpha = .92$). [b] Adult Autism Spectrum Quotient (Baron-Cohen, Wheelwright, Skinner, Martin, & Clubley, 2001).

Measurement of Coordination

To ensure the validity of the frame-differencing approach to measuring interpersonal coordination employed here and better understand precisely what was being measured, we conducted two small follow-up procedures. First, we compared measurements of coordination derived from video with those collected using a magnetic motion-tracking system (Polhemus Liberty; Polhemus Corporation, Colchester, VT) with a tracker attached to the wrist of the dominant hand of each participant. Two small modifications were made to the set-up reported in the main text. First, the distance between the container and the target receptacle was decreased by approximately 30 cm in order to accommodate the size of the area we were able to track using the Polhemus system. Second, we removed the tube and allowed participants to deposit balls into an open container in order to allow for stable in-phase coordination. A pair of participants were then asked to perform three 40 s trials. Initially they were asked to move the balls in time with a metronome (120 bpm) in an anti-phase manner (i.e., when one was picking up a ball the other was depositing), followed by an equivalent but in-phase (i.e., both picking up and depositing at the same time) trial. Finally, participants were told to simply move the balls as quickly as they could, without the metronome or any coordination instructions. For each trial we quantified coordination in terms of cross-spectral coherence from both the video (see main text for details) and motion-tracker recordings, and in terms of relative phase from the motion-tracker recordings only.

Table S3: Summary statistics of comparison between measurements of coordination.

	Video (*FDM*)	Motion-tracking		
Trial	Coherence	Coherence	Relative phase (*M*)	Relative phase (*SD*)
Anti-phase	.62	.85	168.33°	27.03°
In-phase	.63	.89	7.93°	22.22°
No instructions	.21	.04	54.17°	73.48°

As can be seen in Table S3, both measures of cross-spectral coherence were highly consistent but did not distinguish between the in-phase and anti-phase modes of coordination. This data indicates that the movement detected via the video-based frame-differencing approach maps very closely to the task specific arm movements captured by the more fine-grained motion-tracking approach. That more extreme values of coherence were associated with the motion-tracking compared to the video-based analysis likely reflects the greater precision afforded by this approach.

We also conducted a simple rating exercise whereby two independent coders were asked to estimate the relationship between participant movements on a small subset of trials. Specifically, the coders were instructed to use the participant on the left side of the table as a referent and each time she/he deposited a ball to record where in their movement cycle the other participant was according to a 3-point coding scheme: 1 = near the tube (i.e., ≈ in-phase); 2 = midway between the container and the tube (i.e., neither in-phase nor anti-phase); 3 = near the container (i.e., ≈ anti-phase). In total, 20 videos were coded which were quasi-randomly selected such that an equal number of trials from the small and large aperture as well as cooperation and competition conditions were included. We also ensured videos representing a range of coordination levels (coherence = .02 to .86) were included. Comparison between coders revealed a very high degree of consistency for the ‘in-phase’, $r(20) = .87$, $p < .001$, and ‘anti-phase’, $r(20) = .89$, $p < .001$, categories and moderate to high agreement for

the ‘mid-way’ category, $r(20) = .53$, $p = .016$, hence we averaged scores across both coders.[1] Summary percentages are presented in Table S4.

Table S4: Summary of subjective coding of relationship between participants’ movements. The values are ordered by the coherence column (lowest to highest).

			Coding			
Pair	Tube aperture	Condition	Tube	Mid-way	Container	Coherence
18	big	compete	30.6%	26.5%	42.9%	0.024
4	small	compete	5.6%	27.8%	66.7%	0.039
45	big	cooperate	12.2%	23.3%	64.4%	0.052
14	small	compete	26.1%	30.4%	43.5%	0.072
43	big	compete	33.7%	34.7%	31.6%	0.107
6	big	cooperate	30.0%	28.0%	42.0%	0.108
20	small	compete	16.2%	38.4%	45.5%	0.130
27	small	cooperate	0.9%	9.1%	90.0%	0.168
11	big	cooperate	33.3%	23.8%	42.9%	0.186
36	big	compete	21.4%	21.4%	57.3%	0.217
32	small	compete	41.3%	19.6%	39.1%	0.319
16	small	compete	16.3%	30.2%	53.5%	0.369
39	big	cooperate	1.7%	15.7%	82.6%	0.411
29	big	cooperate	17.3%	33.7%	49.0%	0.413
26	small	compete	6.2%	13.3%	80.5%	0.591
3	small	cooperate	3.4%	6.9%	89.7%	0.662
17	big	cooperate	7.3%	26.4%	66.4%	0.678
35	small	cooperate	0.0%	0.0%	100.0%	0.708
9	small	cooperate	1.0%	12.6%	86.4%	0.821
42	big	cooperate	0.0%	0.0%	100.0%	0.858

We then calculated correlations between the percentage counts in each category and estimates of coordination (i.e., cross-spectral coherence) derived from the frame differencing analysis. This revealed a strong positive relationship between the percentage of time participants were judged to be anti-phase (i.e., when one was depositing the other was near to the container) and estimates of coordination, $r(20) = .71$, $p < .001$, and negative relationships for both the ‘mid-way’, $r(20) = - .68$, $p = .001$, and ‘tube’ (i.e., in-phase), $r(20) = - .61$, $p = .004$.

Taken together, both the comparison to the data obtained via the motion-tracking system and to the subjective coding of the relationship between participant movements point to the frame-differencing method we employed to quantify coordination being a valid approach. Estimates of cross-spectral coherence were highly consistent with the motion-tracking data, indicating that we were indeed measuring task-specific movements. In addition, as suspected it also appears that the coordination observed was primarily of an anti-phase mode.

[1] It should be noted that this pattern of more accurate judgements of coordination being associated with the stable phase modes (i.e., in-phase and anti-phase) is consistent with the extant literature (e.g., Lumsden, Miles & Macrae, 2012; Zaal, Bingham, & Schmidt, 2000). Moreover, the angle from which the video was recorded made precise establishment of the physical boundaries for classification into the intermediary category difficult, leading to the expectation of more consistency for the in-phase and anti-phase categories.

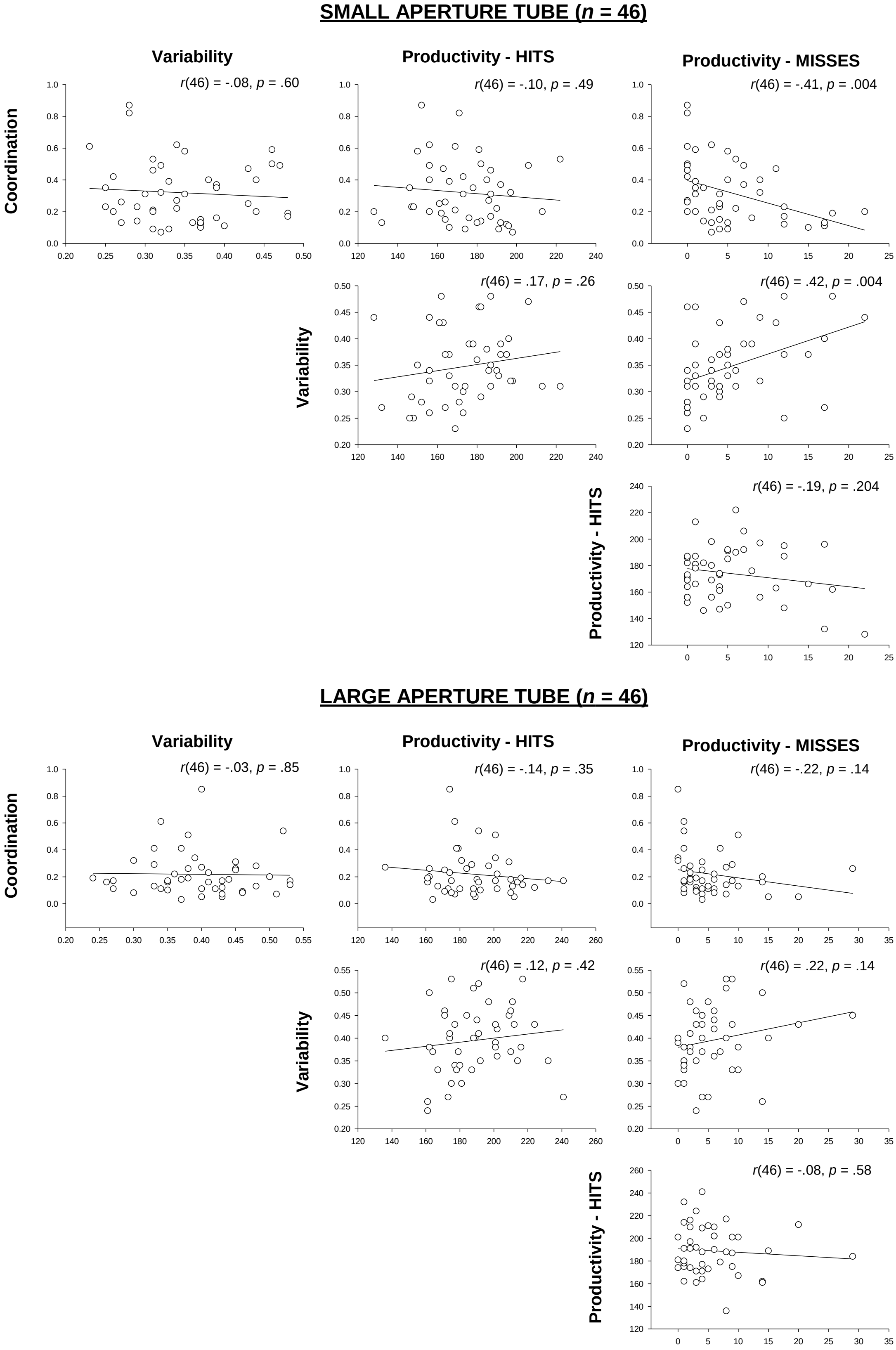

Figure S1: Matrix of scatterplots showing the linear relationships between coordination (i.e., cross-spectral coherence), movement variability (i.e., coefficient of variation), hits and misses for the small and large apertures separately. Table 1 in the main text presents the correlation coefficients.

Supplemental Materials – References

Baron-Cohen, S., Wheelwright, S., Skinner, R., Martin, J., & Clubley, E. (2001). The autism spectrum quotient (AQ): Evidence from Asperger syndrome/high functioning autism, males, females, scientists and mathematicians. *Journal of Autism and Developmental Disorders*, *31*, 5-17.

Fitzpatrick, P., Frazier, J. A., Cochran, D. M., Mitchell, T., Coleman, C., & Schmidt, R. C. (2016). Impairments of social motor synchrony evident in autism spectrum disorder. *Frontiers in Psychology*, *7*, 1323.

Liebowitz, M. R. (1987). Social phobia. *Modern Problems of Pharmacopsychiatry*, *22*, 141-173.

Lumsden, J., Miles, L. K., & Macrae, C. N. (2012). Perceptions of synchrony: Different strokes for different folks? *Perception*, *41*, 1529-1531.

Lumsden, J., Miles, L. K., Richardson, M. J., Smith, C. A., & Macrae, C. N. (2012). Who syncs? Social motives and interpersonal coordination. *Journal of Experimental Social Psychology*, *48*, 746-751.

Van Lange, P. A. M. (1999). The pursuit of joint outcomes and equality in outcomes. An integrative model of social value orientation. *Journal of Personality and Social Psychology*, *77*, 337-349.

Varlet, M., Marin, L., Capdevielle, D., Del-Monte, J., Schmidt, R. C., Salesse, R. N., Boulenger, J-P., Bardy, B. G., & Raffard, S. (2014). Difficulty leading interpersonal coordination: Towards an embodied signature of social anxiety disorder. *Frontiers in Behavioral Neuroscience*, *8*, 29.

Zaal, F. T. J. M., Bingham, G. P., & Schmidt, R. C. (2000). Visual perception of mean relative phase and phase variability. *Journal of Experimental Psychology: Human Perception and Performance*, *26*, 1209-1220.
